# Bipolar radiofrequency ablation is useful for treating atrial fibrillation combined with heart valve diseases

**DOI:** 10.1186/1471-2482-14-32

**Published:** 2014-05-22

**Authors:** Lin Chen, Yingbin Xiao, Ruiyan Ma, Baicheng Chen, Jia Hao, Chuan Qin, Wei Cheng, Renguo Wen

**Affiliations:** 1 Department of Cardiovascular Surgery, Xinqiao Hospital, Third Military Medical University, 7F, Second in-Patient Building, 183 Xinqiao St, Shapingba District, Chongqing 400037, P. R. China

**Keywords:** Radiofrequency ablation, Bipolar, Atrial fibrillation

## Abstract

**Background:**

Atrial fibrillation (AF) is a common arrhymia, and it results in increased risk of thromboembolism and decreased cardiac function. In patients undergoing cardiac surgery, concomitant radiofrequency ablation to treat AF is effective in restoring sinus rhythm (SR). This study is an observational cohort study aimed to investigate the safety and efficacy of bipolar radiofrequency ablation (BRFA) for treating AF combined with heart valve diseases.

**Methods:**

Clinical data were analyzed retrospectively from 324 cases of rheumatic heart disease combined with persistent AF patients who underwent valve replacement concomitant BRFA. The modified left atrial and the simplified right atrial ablation were used for AF treatments. Of the 324 patients, 248 patients underwent mitral valve replacement and 76 patients underwent double valve replacement. Meanwhile, 54 patients underwent concomitant thrombectomy and 97 underwent tricuspid valvuloplasty. And all of them received temporary pacemaker implantation. The 24 hours holter electrocardiogram (ECG) monitoring and echocardiography was performed before the operation, on the first day after operation, on discharge day, and at 6 and 12 months after operation.

**Results:**

There were 299 patients with SR on the first day after operation (92.30%), 12 patients with junctional rhythm (3.70%), 11 patients with AF (3.39%), and 2 patients with atrial flutter (0.62%). The temporary pacemaker was used in 213 patients (65.74%) with heart rates less than 70 beat/minute in the ICU. Two patients died early and the mortality rate was 0.62%. Two patients had left ventricular rupture and the occurrence rate was 0.62%. They both recovered. There was no degree III atrioventricular blockage and no permanent pacemaker implantation. Overall survival rate was 99.38% (322 cases) with SR conversion rate of 89.13% (287 cases) at discharge. The SR conversion rate was 87.54% and 87.01% at 6 and 12 months after operation. Sinus bradycardia occurred in 3.42% of patients at 6 months after operation and in 3.03% of patients at 12 months after operation. Echocardiography showed that the left atrial diameter was significantly decreased, and ejection fraction and fractional shortening were significantly improved.

**Conclusions:**

BRFA for treating AF in concomitant valve replacement is safe and with good efficacy.

## Background

Atrial fibrillation (AF) is a common cardiac arrhythmia, and frequently complicates the course of heart valve disease [1-3]. Many patients who receive mitral valve replacement (MVR) have AF, and many other of them develop AF after the surgery [2]. The efficacy of internal medicine therapy and electric cardioversion is not satisfactory and thus their clinical application is limited [3]. With the increasing application of electrophysiological mapping, radiofrequency ablation in the treatment of cardiac arrhythmia is in rapid progress. Radiofrequency ablation is the breakthrough for the treatment of chronic AF. In patients undergoing cardiac surgery, concomitant radiofrequency ablation to treat AF is effective in restoring sinus rhythm (SR) [4-7].

Mccarthy et al. [8] applied 5 different methods for concurrent treatment of AF in 408 cases of heart operation. They found that the cure rate for patients received strong focused ultrasound ablation was only 43% in the one-year follow up time. Cox-maze procedure is the classic and effective surgical treatment for AF, with a high success rate of 90%. On the contrary, traditional cut-and-sew technique is time-consuming and difficult with complex operation incision and high rate of postoperative complications (nearly 23% of patients had postoperative implantation of permanent pacemaker), and thus its application is limited [9]. In addition, the success rate for pulmonary vein ablation is 69%, while the success rate for left atrial maze ablation and biatrial maze ablation both are 79%, which indicates the significant efficacy of atrial maze ablation.

In this study, the safety and efficacy of left atrial and simplified right atrial maze ablation for the treatment of persistent AF in concomitant valve replacement were analyzed by using Atricure™ bipolar radiofrequency ablation (BRFA) apparatus.

## Methods

### General information

From September 2009 to June 2012, 3360 consecutive patients underwent heart valve replacement for rheumatic heart disease at Xinqiao Hospital, Third Military Medical University, ChongQing, China. Of the 3360 patients, 324 patients underwent valve replacement and concurrent BRFA for treatment of persistent AF. Among them, there were 136 males and 188 females, aging from 28 to 71 years old. They had clinical presentations like angina and shortness of breath, with AF history for 1 year to 15 years. According to 2011 ACCF/AHA/HRS and 2013 EACTS Guidelines [10,11], they all had long-persistent AF. Preoperatively they were confirmed of persistent AF by 12 lead electrocardiogram (ECG) examination and 24 hour holter ECG monitoring. There were 86 cases in NYHA functional class I to II, 182 in class III, and 56 in class IV. Echocardiography showed that the left atrial diameter (LAd) was 39 to 85 mm, left ventricular diameter (LVd) was 41 to 75 mm, and left ventricular ejection fraction (LVEF) was 40% to 67%. For patients with heart rate less than 60 beat/min, or with LAd more than 85 mm, or received operation before, or with severe pericardial adhesions, concomitant BRFA were not performed.

Prior written and informed consent were obtained from every patient and the study was approved by the ethics review board of Xinqiao Hospital, Third Military Medical University.

### Surgical approaches

**Figure 1:**
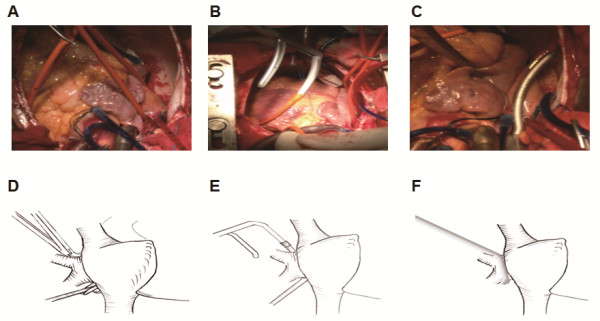
**Radiofrequency ablation by a urine catheter-guided bipolar radiofrequency clamp technique. (A)** Left and right pulmonary veins were separated and circled with a urine catheter. **(B)** The bipolar radiofrequency clamp arm was inserted into the catheter. **(C)** The bipolar radiofrequency clamp was directed by the catheter to go through the back wall of the left and right pulmonary veins. Then the circled ablation of the pulmonary veins was performed. **(D)** Diagram of surgery shown in **A**. **(E)** Diagram of surgery shown in **B**. **(F)** Diagram of surgery shown in **C**.

Operations were performed under general anesthesia with hypothermic cardiopulmonary bypass (CPB). Atricure™ BFRA apparatus was applied as the method described by Sims et al. [1] and was adjusted according to the condition of the patients. Briefly, the left and right pulmonary vein (PVs) were carefully separated and encircled with a urine catheter. With the guidance of the catheter, the bipolar ablation clamp was positioned precisely around the PVs. Circle ablation of the right and left PVs was performed (see Figure 1). After Marsh ligaments cutting and cross-clamp the ascending aorta, left atrial cavity was exposed through a left atrial incision behind the interatrial groove. Then the linear ablations were performed between the left and right inferior PVs, between the left and right superior PVs, and between the left superior PV and the opening of left atrial appendage. The maze ablation was repeated 4-5 times during circle ablation of the right and left PVs, during the linear ablation between the left and right inferior PVs, and during the linear ablation between the left and right superior PVs. Then, the orifice of left atrial appendage was closed with continuous suture, followed by valve replacement and the ablation of the right atrium.

For patients with obviously enlarged left atrium, interatrial groove incision was performed for MVR. For patients with severe tricuspid regurgitation, transseptal approach was conducted for MVR. Aortic replacement procedure (AVR) was performed through a incision of ascending aorta. Both MVR and AVR were done in interrupted suture in all the patients. After MVR, the ablation of the right atrium was performed. The linear ablations were performed in the regions from the vertical incision on anterior wall of the right atrium to the interatrial groove and to the tricuspid annulus; and, from the lower edge of the incision to the coronary sinus ostium and to the inferior vena cava; and, from the lower edge of the coronary sinus ostium to the inferoseptal commissure.

### Postoperative treatment and follow up

Holter ECG monitoring for 24 hours and echocardiography were performed before the operation, on the first day after operation, on discharge day, and at 6 months and 12 months after operation. AF recurrence was defined as the appearance of AF in the 24 hours holter ECG monitoring. During ICU, patients were treated with intravenous amiodarone infusion. After they were transferred out of ICU, they were treated with oral administration of 200 mg amiodarone twice each day. When they were discharged, oral administration of 200 mg amiodarone was given once per day for 3-6 months.

### Statistical analysis

SPSS V11.0 statistical software was applied for statistical analysis. Data was presented as $\bar{x}$ ± s, and categorical data was examined by X^2^ test. P < 0.05 was considered significantly different.

## Results

As shown in Table 1, among the 324 patients, 248 patients underwent BRFA and concomitant MVR, and 76 patients underwent double valve replacement (DVR). Meanwhile, 54 patients underwent concomitant thrombectomy, and 97 patients underwent tricuspid valve annuloplasty (including 33 cases using Cosgrove Edwards annuloplasty ring). All patients received prophylactic temporary pacemaker implantation. In ICU, when the heart rate was less than 70 beat/minute, the temporary pacemaker was used.

**Table 1:** **General surgical information (**$\bar{x}$**± s)**

**Variable**	**n (%) or**$\bar{x}$**± s**
Gender (male/female, case)	136 (42.00)
Age (year)	50.67 ± 18.33
Mitral valve replacement (case)	248 (76.54)
Double valve replacement (case)	76 (23.46)
Concomitant tricuspid valve annuloplasty (case)	97 (29.94)
Concomitant thrombectomy (case)	54 (16.67)
Cardiopulmonary bypass time (min)	106.80 ± 25.72
Aortic occlusion time (min)	65.91 ± 20.01
Ventilation time (h)	20.70 ± 7.37
Chest tube drainage (ml)	358.11 ± 137.38

A total of 299 patients had SR on the first day after operation (92.30%), 12 patients had junctional rhythm (3.70%), 11 patients had atrial fibrillation (3.39%), and 2 patients had atrial flutter (0.62%). In ICU, 213 patients used temporary pacemaker (65.74%). Two patients died early and the mortality rate was 0.62%. One of them died the day after operation because of pulmonary hypertension crisis, and the other died 29 days after the operation because of multiple organ failure. There were 2 cases of left ventricle rupture, with an occurrence rate of 0.62%. One left ventricle rupture appeared 8 hours after transferred to ICU and the other left ventricle rupture appeared as soon as weaning off CPB. After treatment, they were both recovered. Early operation complications and mortality rates were shown in Table 2. The echocardiography changes before and after operation were shown in Table 3. Echocardiography showed that the left atrial diameter was significantly decreased, and ejection fraction and fractional shortening were significantly improved.

**Table 2:** Early complications and mortality rates perioperatively (%)

**Variable**	**n (%)**
Complications	
Repeat thoracotomy	4 (1.23%)
Low cardiac output syndrome	9 (2.78%)
Respiratory failure	12 (3.70%)
Kidney failure	3 (0.93%)
Multiple organ failure	4 (1.23%)
Left atrium rupture	2 (0.62%)
Cerebral hemorrhage	1 (0.31%)
Early death	2 (0.62%)

**Table 3:** **Echocardiography change before and after the operation (**$\bar{x}$**± s)**

**Groups**	**Diameter of left artrium (mm)**	**Ejection fraction (%)**	**Fractional shortening (%)**
Before operation (n = 324)	57.48 ± 15.16	56.57 ± 9.67	33.47 ± 7.08
Before discharge (n = 322)	38.81 ± 15.14^##^	59.30 ± 11.32	35.18 ± 6.67^#^
Six months after operation (n = 322)	36.94 ± 10.23^##^	62.35 ± 13.61^#^	34.53 ± 7.64
Twelve months after operation (n = 231)	37.66 ± 12.01^##^	64.01 ± 12.33^#^	35.73 ± 6.18^#^

Note: ^#^compare with before operation; p < 0.05; ^##^*p* < 0.01.

There was no degree III atrioventricular blockage. Overall survival rate was 99.38% with SR conversion rate of 89.13% at discharge. The SR conversion rate was 87.54% and 87.01% at 6 and 12 months of follow-up. Sinus bradycardia, with heart beat ranging from 50-58 beat/minute, occurred in 3.42% of patients at 6 months after operation and in 3.03% of patients at 12 months after operation. They had no chief complaint, and therefore there were no permanent pacemaker implantation. The ECG changes before and after operation were shown in Table 4.

**Table 4:** Electrocardiogram change before and after the operation (%)

**Groups**	**Sinus rhyme**	**Junctional rhyme**	**Atrial fibrillation**	**Atrial flutter**	**Sinus bradycardia**
The day after operation (n = 324)	299 (92.30)	12 (3.70)	11 (3.40)	2 (0.62)	91 (28.09)
Before discharge (n = 322)	287 (89.13)	8 (2.48)	17 (5.24)	10 (3.09)	9 (2.80)*
Six months (n = 322)	282 (87.58)*	7 (2.17)	17 (5.27)	16 (4.97)*	11 (3.42)*
Twelve months (n = 231)	201 (87.01)*	7 (3.03)	12 (5.19)	11 (4.76)*	7 (3.03)*

Note: *compare with the day after operation, p < 0.05.

## Discussion

In this study, Atricure™ BRFA instrument was applied for left atrial and simplified right atrial maze ablation according to the method of Sims et al. [1] with minor modifications. Compared with unipolar flushing type radiofrequency ablation, Atricure™ BRFA instrument is with simpler operation method and better atrial transmural ablation. And, through using Atricure™ BRFA instrument, it is easier to determine the transmural ablation time and effect, and more effective to avoid the cardiac damage [1,12-14]. The BRFA system is designed based on the stable and good penetration properties of radiofrequency current. The local hyperthermia effect induced by the radiofrequency current can produce tissue coagulation necrosis and thus AF reentrant loop is blocked [1]. Schuessler et al. [15] summarized the use of nine different unidirectional devices (including four radiofrequency, two microwave, two lasers and one cryothermic) for creating continuous transmural lines of ablation from the atrial epicardium in a porcine model. They defined a unidirectional device as one in which all the energy was applied by a single transducer on a single heart surface. The maximum penetration of any device was 8.3 mm. All devices except one, the AtriCure Isolator pen, failed to penetrate 2 mm in some non-transmural sections. Melby et al. reported that bipolar ablation of different atrial structures required widely different amounts of energy and ablation times, probably as the result of the inhomogeneity of atrial geometry and tissue impedance [16]. In this study, the ablation was repeated 4-5 times during circle ablation of the right and left PVs, the linear ablation between the left and right inferior and the linear ablation between the left and right superior PVs. For performing the procedure of maze ablation, extra 10 to 15 minutes was required for cross-clamp and extra 20 to 35 minutes for bypass. Our results were consistent with previous findings. Von Oppell et al. reported that an average of 30 minutes extra was required both for bypass and cross-clamp when performing the procedure of maze ablation [4]. The efficacy of BRFA in the present study was satisfactory and 92.30% of patients had SR on the first day after operation. Overall survival rate was 99.38% with SR conversion rate of 89.13% on discharge day. The SR conversion rate was 87.54% at 6 months after operation, and 87.01% at 12 months after operation. Echocardiography showed that the left atrial diameter was significantly decreased, and ejection fraction and fractional shortening were significantly improved. The results were very good, and it was similar to the reported guideline [10]. Dunninga et al. [10] reported that BRFA, as an adjunct to cardiac surgery, had a higher success rate in restoring SR compared with no ablation for at least 1 year. Success rates ranged from 54% to 90% after a medium-term follow-up for at least 12 months. There is limited evidence suggesting superiority of BRFA over unipolar energy as yet [10].

Since the occurrence rate of sinus bradycardia is higher at early postoperative, all patients received prophylactic temporary pacemaker implantation in this study. Sinus bradycardia occurred in 91 patients (28.09%) during ICU. Postoperative patients with sinus bradycardia usually recovered around 3 days. During follow-up, sinus bradycardia rate was 3.42% at 6 months and 3.03% at 12 months with heart rate of 50–60 beat/minutes, and there were no complaints and no permanent pacemaker implantation.

In this study, the percentage of patients with early postoperative respiratory failure was 3.7%, which was relatively high. Increased lung injury may be caused by the following reasons. During the BRFA procedure, there was a short cross-clamp of the left and right superior PVs. This may result in a sharp increase in pulmonary venous pressure and cause injury to the pulmonary capillary and the endothelial cell. In addition, the thermal reaction during BRFA could lead to regional myocardial and PVs injury, which may result in systemic inflammatory response syndrome [1]. Thus, we suggest that ventilator support should be properly prolonged after operation for patients with long time left and right pulmonary vein isolation.

Rupture of left ventricle is one of the rare and most serious complications of MVR. It is difficult to treat and can cause high mortality rate [17]. There were 2 patients of left ventricular rupture in this study. Both of them were old female with severe mitral stenosis combined with AF. This indicates that left ventricular rupture may be one of the risks during performing MVR concomitant BRFA in older women with AF and severe mitral stenosis. We suppose that BRFA may injure the left ventricle under mitral annulus by mistake during performing the lineal ablation from interatrial groove incision margin to posterior mitral valve annulus. And to perform MVR concomitant BRFA for treating AF, the heart has to be moved and lifted repeatedly. It might increase the possibility of atrioventricular dehiscence when the heart is repeatedly moved and lifted during heart beating in old female with severe stenosis of rheumatic mitral [17]. We suggest that during performing MVR concomitant BRFA for AF, more attention should be paid to the linear ablation from interatrial groove incision margin to posterior mitral valve annulus. We recommend the urine catheter guided BRFA clamp technique when performing MVR concomitant BRFA. Compared with the bipolar forceps, dissociative clamp has short operating handle and appropriate radian. Dissociative clamp of the appropriate size can be chosen according to the size of heart. It is of great flexibility and is easy to control. With dissociative clamp, the procedure is simplified and the surgical trauma and risks are reduced during separating the left and right pulmonary veins. Meanwhile, when the left atrial is significantly enlarged and pulmonary vein is widened, arm of bipolar radiofrequency forceps might be too short to fully cover pulmonary vein. Traction the urine catheter could support the bipolar radiofrequency clamp to completely cover the pulmonary vein. When the catheter is removed, ablation pulmonary vein could be more thorough. Ordinary catheter is convenient to be obtained since materials are cheap. Catheter is soft, smooth, and flexible, so the cardiac tissue will not be injured.

## Conclusions

In conclusion, the efficacy of modified left atrial and simplified right atrial maze ablation by BRFA for the treatment of AF is satisfactory. The procedure is a safe, feasible, effective and simple surgical technique. However, its long-term effects still need further study.

### Limitation

The present study is a retrospective and nonrandomized study. Furthermore, follow-up duration is relatively short, and a longer follow-up are required.

## Abbreviations

ACCF: American College of Cardiology Foundation; AF: Atrial Fibrillation; AHA: American Heart Association; AVR: Aorta valve replacement; BRFA: Bipolar Radiofrequency Ablation; CPB: Cardiopulmonary bypass; DVR: Double valve replacement; EACTS: European Association of Cardiothoracic Surgery; ICU: Intensive Care Unit; LAd: Left atrial diameter; LVd: Left ventricular diameter; LVEF: Left ventricular ejection fraction; MVR: Mitral valve replacement; PV: Pulmonary vein; SR: Sinus rhythm.

## Competing interests

The authors declare that they have no competing interests.

## Authors’ contributions

LC was the senior author of the paper, performed all surgical procedures, and was the primary writer of the manuscript. YBX conceived the study, participated in the design and coordination of the study, and helped to draft the manuscript. BCC and RGW participated in patient care, spoke with the patients, and contributed significantly to the intellectual content of the study. RYM and JH conducted an extensive literature search. WC and CQ participated in the design of the study and performed the statistical analysis. All authors read and approved the final manuscript.

## Pre-publication history

The pre-publication history for this paper can be accessed here:

http://www.biomedcentral.com/1471-2482/14/32/prepub

## References

[B1] Sims JB Roberts WC Comparison of findings in patients with versus without atrial fibrillation just before isolated mitral valve replacement for rheumatic mitral stenosis (with or without associated mitral regurgitation) Am J Cardiol 2006 97 1035 1038 10.1016/j.amjcard.2005.11.023 16563911

[B2] Hussein AA Wazni OM Harb S Joseph L Chamsi-Pasha M Bhargava M Martin DO Dresing T Callahan T Kanj M Natale A Lindsay BD Saliba WI Radiofrequency ablation of atrial fibrillation in patients with mechanical mitral valve prostheses J Am Col Cardiol 2011 58 596 602 10.1016/j.jacc.2011.03.039 21798422

[B3] Cannom DS Atrial fibrillation: nonpharmacologic approaches Am J Cardiol 2000 85 25D 35D 10.1016/S0002-9149(00)00904-8 10822038

[B4] von Oppell UO Masani N O'Callaghan P Wheeler R Dimitrakakis G Schiffelers S Mitral valve surgery plus concomitant atrial fibrillation ablation is superior to mitral valve surgery alone with an intensive rhythm control strategy Euro J Cardio-Thorac Surg 2009 35 641 650 10.1016/j.ejcts.2008.12.042 19233678

[B5] Kim JB Yun TJ Chung CH Choo SJ Song H Lee JW Long-term outcome of modified maze procedure combined with mitral valve surgery: analysis of outcomes according to type of mitral valve surgery J Thora Cardiovasc Surg 2010 139 111 117 10.1016/j.jtcvs.2009.07.002 19740489

[B6] Lakkireddy D Nagarajan D Di Biase L Vanga SR Mahapatra S Jared Bunch T Day JD Burkhardt DJ Umbarger L Dendi R Pimentel R Berenbom L Emert M Gerken A Bommana S Ray W Atkins D Murray C Dawn B Natale A Radiofrequency ablation of atrial fibrillation in patients with mitral or aortic mechanical prosthetic valves: A feasibility, safety, and efficacy study Heart Rhythm 2011 8 975 980 10.1016/j.hrthm.2011.02.012 21316485

[B7] Schulenberg R Antonitsis P Stroebel A Westaby S Chronic atrial fibrillation is associated with reduced survival after aortic and double valve replacement Ann Thorac Surg 2010 89 738 744 10.1016/j.athoracsur.2009.12.023 20172119

[B8] McCarthy PM Kruse J Shalli S Ilkhanoff L Goldberger JJ Kadish AH Arora R Lee R Where does atrial fibrillation surgery fail? Implications for increasing effectiveness of ablation J Thorac Cardiovas Surg 2010 139 860 867 10.1016/j.jtcvs.2009.12.038 20304134

[B9] Cox JL Ad N Palazzo T Fitzpatrick S Suyderhoud JP DeGroot KW Pirovic EA Lou HC Duvall WZ Kim YD Current status of the maze procedure for the treatment of atrial fibrillation Semin Thorac Cardiovase Surg 2000 12 15 19 10.1016/s1043-0679(00)70011-6 10746917

[B10] Dunninga J Nagendranb M Alfieric OR Eliad S Kappeteine AP Lockowandtf U Sarrisg GE Kolhh PH EACTS guideline for the surgical treatment of atrial fibrillation Eur J Cardiothorac Surg 2013 44 777 791 10.1093/ejcts/ezt413 23956274

[B11] Wann LS Curtis AB January CT Ellenbogen KA Lowe JE Estes NA III Page RL Ezekowitz MD Slotwiner DJ Jackman WM Stevenson WG Tracy CM ACCF/AHA/HRS focused update on the management of patients with atrial fibrillation (updating the 2006 guideline): a report of the American College of Cardiology Foundation/American Heart Association Task Force on Practice Guidelines Circulation 2011 2011 123 104 123 21173346 10.1161/CIR.0b013e3181fa3cf4

[B12] Melby SJ Zierer A Bailey MS Cox JL Lawton JS Munfakh N Crabtree TD Moazami N Huddleston CB Moon MR Damiano RJ Jr A new era in the surgical treatment of atrial fibrillation: the impact of ablation technology and lesion set on procedural efficacy Ann Surg 2006 244 583 592 16998367 10.1097/01.sla.0000237654.00841.26 PMC1856555

[B13] Srivastava V Kumar S Javali S Rajesh TR Pai V Khandekar J Agrawal N Patwardhan AM Efficacy of three different ablative procedures to treat atrial fibrillation in patients with valvular heart disease: a randomised trial Heart Lung Circ 2008 17 232 240 10.1016/j.hlc.2007.10.003 18242137

[B14] Basu S Nagendran M Maruthappu M How effective is bipolar radiofrequency ablation for atrial fibrillation during concomitant cardiac surgery? Interact Cardiovasc Thorac Surg 2012 15 741 748 10.1093/icvts/ivs311 22815321 PMC3445386

[B15] Schuessler RB Lee AM Melby SJ Voeller RK Gaynor SL Sakamoto S Damiano RJ Animal studies of epicardial atrial ablation Heart Rhythm 2009 6 12 Suppl S41 S45 19959142 10.1016/j.hrthm.2009.07.028 PMC2907672

[B16] Melby SJ Zierer A Voeller RK Lall SC Bailey MS Moon MR Schuessler RB Damiano RJ Jr Wide variations in energy delivery using an impedance-controlled algorithm in bipolar radiofrequency ablation: evidence against fixed time ablation Innovations 2007 2 67 72 22436925 10.1097/IMI.0b013e31803c9b11

[B17] Deniz H Sokullu O Sanioglu S Sargin M Ozay B Ayoglu U Aykut Aka S Bilgen F Risk factors for posterior ventricular rupture after mitral valve replacement: results of 2560 patients Eur J Cardiothorac Surg 2008 34 780 784 10.1016/j.ejcts.2008.06.009 18621539

